# Association of Salt-Reduction Knowledge and Behaviors and Salt Intake in Chinese Population

**DOI:** 10.3389/fpubh.2022.872299

**Published:** 2022-04-18

**Authors:** Bing Han, Chuancang Li, Yabing Zhou, Mengge Zhang, Yang Zhao, Ting Zhao, Dongsheng Hu, Liang Sun

**Affiliations:** ^1^Section of Chronic and Noncommunicable Diseases Prevention and Control, Henan Provincial Center for Disease Control and Prevention, Zhengzhou, China; ^2^Department of Social Medicine and Health Management, School of Public Health, Zhengzhou University, Zhengzhou, China; ^3^Department of Epidemiology and Biostatistics, School of Public Health, Zhengzhou University, Zhengzhou, China

**Keywords:** salt-reduction, knowledge, behaviors, Chinese population, salt intake

## Abstract

**Objective:**

Excessive salt intake is causally associated with an increased risk of cardiovascular disease. Salt-reduction strategies have been rapidly deployed across China since 2017. This study aimed to investigate the association of salt-reduction knowledge and behaviors and salt intake in Chinese population.

**Study Design:**

This study was a national cross-sectional study in China.

**Methods:**

This cross-sectional study was based on data collected during a Chinese adult chronic disease and nutrition surveillance program in 2018 with 7,665 study participants. Salt intake was assessed by calculating 24 h urine sodium from morning urine samples. Logistic regression and mean impact value (MIV) based on the back propagation (BP) artificial neural network were used to screen the potential influencing factors.

**Results:**

A total of 7,665 participants were included in the analysis, with an average age of 54.64 ± 13.26 years, and with men accounting for 42.6%. Only 19.3% of the participants were aware of the Chinese Dietary Guidelines, and only 7.3% of them could accurately identify the level of salt intake recommended in the Chinese Dietary Guidelines. Approximately 41% of the participants adopted salt-reduction behaviors, among whom the number of participants who used less salt when cooking was the highest, and the number of participants who used low sodium salt was the lowest. In the logistic regression, only “No extra salt was added at the table” group showed the effect of salt-reduction, the odds ratio (OR) being 0.78 (95% confidence interval [CI]: 0.64–0.95). The MIV result based on the BP neural network showed that the most important salt-reduction behavior was using less salt when cooking, while reducing eating-out behavior and using salt-limiting tools were the least important.

**Conclusion:**

The research shows that the popularization of salt-reduction knowledge and behaviors can reduce the population's salt intake. However, there is still considerable scope for promoting salt-reduction knowledge and behaviors, while the promotion of salt-reduction tools and low-sodium salt still needs to be strengthened.

## Introduction

Salt, namely sodium chloride, is a common and important condiment in people's daily lives. Sodium is a very important nutrient that participates in many important physiological processes. A large number of epidemiological evidences illustrate that excessive salt intake is harmful to health, and with mean systolic blood pressure increasing by 2.86 mmHg per 1 g increase in mean sodium intake, it is not surprising that the occurrence of hypertension is closely related to it (1–5). Hypertension is a major risk factor for cardiovascular disease, chronic kidney damage, and death (6–8). In the 2015 Global Burden of Disease Study (9), high dietary sodium intake was identified as one of the top 10 risk factors leading to global disability-adjusted life years (DALY).

The World Health Organization (WHO) argues that the population nutrient intake goal for salt should be no more than 5 g/day (10). In China, the Chinese Nutrition Society recommends a salt intake of no more than 6 g/day based on the characteristics of Chinese adults (11), but Chinese residents traditionally have a high salt diet. According to the INTERMAP study, the salt intake of the Chinese population reached 13.3 g/day (12), causing the Chinese government to declare reducing salt intake as a goal in health policy in China. In 2017, the State Council set a goal of reducing the national salt intake by 20% by 2030 (13–15). Salt-reduction strategies have been rapidly implemented across China. Local governments such as those in Beijing, Shanghai, and Shandong also organized salt-reduction projects in many of their provinces and cities (16, 17).

With the constant promotion of salt-reduction projects in China, salt intake has been declining in many provinces, with the concept of salt-reduction being accepted by the public. This study aimed to study the effects of salt-reduction projects in Henan Province, China and to evaluate the association between salt-reduction knowledge and behaviors and salt intake.

As we all know, there is a complicated association between behavioral factors and salt intake that is difficult to evaluate with conventional statistical methods. The artificial neural network (ANN), however, is characterized by non-linearity, self-adaptation, and parallel processing (18), while mean impact value (MIV) is considered to be one of the most reliable tools for evaluating variable correlation in neural networks. We suggest that the ANN model can reveal the complex relationship between salt-reduction behaviors and salt intake by ranking salt-reduction behaviors.

## Methods

### Study Population

This study was a cross-sectional study. Data was collected from the China Adult Chronic Disease and Nutrition Surveillance Program in Henan province, a program conducted from November 2017 to June 2019. Between 2015 and 2019, the National Health Commission launched a new round of chronic disease and nutrition surveillance of Chinese residents in 31 provinces across the country. 8,710 people were investigated in Henan Province. The subjects of this survey were permanent residents in the monitoring sites, with “permanent residents” being defined as Chinese residents who had lived in the monitored area for more than 6 months in the 12 months before the survey. The inclusion criteria for the study included adults aged between 18 and 79 years with an estimated glomerular filtration rate (eGFR) of at least 60 ml/min per 1.73 m^2^. Individuals with the following conditions were excluded: (1) those with severe chronic kidney diseases such as nephritis, diabetic nephropathy, hypertensive renal damage, and polycystic kidney; (2) female participants who were pregnant; and (3) residents with cognitive impairment, serious illness, or disability that could affect the investigation. The final analysis sample included 7,665 respondents. All participants provided written informed consent.

### Sample

A multi-stage stratified cluster sampling method was used to sample from 14 monitoring points in 12 cities in Henan Province. In the first stage, within the monitoring points, a systematic sampling of population size ranking was conducted, with 3 townships randomly selected. In the second stage, among the randomly selected townships, a systematic sampling according to population size was conducted, with 2 villages (residential committees) randomly selected. In the third stage, a simple random sampling method was used to collect 1 villager group, which consisted of 60 or more families from the 2 villages that were randomly selected. In the fourth stage, 45 families were selected from the selected villager groups to participate in the survey.

### Data Collection

Data were collected by a personal questionnaire, by physical measurement, and in laboratory examination. The questionnaire mainly included the participants' personal information, their awareness of dietary salt intake, their salt-reduction behaviors, and their eating habits. Respondents were asked to fast for 8 to 12 h. The measurement methods were in line with the requirements of the China Health Monitoring Body Measurement Methods industry standard (WS/T424-2013). Height measurement was taken with the TZG sitting height meter (Hongya, Wuxi/China), while the body weight measurement was performed with the TC-200K electronic weight scales (G&G, Changshu/China). The same instruments, used in all monitoring sites, had been inspected by the quality inspection department. All the investigators participated in similar training and examination sessions, and only those who passed the sessions were able to participate in the survey work. Respondents were asked to provide 5 ml of morning urine, by collecting it in the urine cup provided in advance and returning it to the investigator while filling the personal questionnaire. The collected urine was tested by a qualified central laboratory that had passed the quality assessment of the national project working group. Urine creatinine was tested using the enzyme-coupled sarcosine oxidase method, while urine sodium was tested using the ion-selective electrode method.

### Salt Intake Assessment

Salt intake assessment methods included 24-h diet recall, completion of a food frequency questionnaire, and urine assessment. The 24-h urinary sodium (24-hUNa) method is the widely-accepted gold standard for evaluating individual dietary salt intake (19). It is difficult to perform a complete 24-h urine collection, so this study used the simple estimation method developed by Professor Kawasaki (20) in 1993 to estimate 24-hUNa excretion using morning urine specimens. The formula estimates 24-hUNa excretion based on spot urinary sodium (spot Na), spot creatinine (spot Cr), and prediction creatinine (Pre Cr). Pre Cr was calculated based on height, weight, and age of participants. Salt intake was calculated using the 24-hUNa. This estimation method has been validated in the Chinese population and has the advantage of smaller deviations compared to other estimation methods in predicting the dietary salt intake of a population (21, 22).


$$\begin{array}{l} 24\;-hUNa:\;(mg/day)=23\times 16.3\times \{[spot\;Na(mmol/L)\\ \;\;÷spot\;Cr(mg/dL)\times 10]{\times Pre\;Cr(mg/day)\}}^{0.5} \end{array}$$

$$\begin{array}{l} Men:\;Pre\;Cr\left(mg/day\right)=15.2\times weight\left(kg\right)+7.39\times height\left(cm\right)\\ -12.63\times age\left(years\right)-79.9 \end{array}$$

$$\begin{array}{l} Women:\;Pre\;Cr\left(mg/day\right)=8.58\times weight\left(kg\right)+5.09\\ \times height\left(cm\right)-4.72\times \;age\left(years\right)-74.95 \end{array}$$

### Statistical Analysis

Continuous variables were described by mean ± standard deviation, while categorical variables were described by percentage. The *t*-test was used to compare the difference of salt intake between the participants, according to salt-reduction awareness, knowledge, and behaviors, respectively.Participants were divided into two groups according to their salt intake.The high intake group was >10.5 g/day, and the low intake group was ≤ 10.5 g/day. The association between salt-reduction behaviors and salt intake was explored by logistic regression. The MIV based on the BP artificial neural network was used to evaluate the impact of each test behavior on salt intake. Statistical analysis was performed using IBM SPSS21. All analyses were two-tailed, and a *p*-value of <0.05 was considered statistically significant. Based on the MATLAB (MathWorks, Natick, MA), the ANN prediction model was established, and each input neuron's MIV could be calculated.

## Results

The baseline characteristics of participants are shown in Table 1. A total of 7,665 participants were included in this study, including 3,269 males, accounting for 42.6% of all surveyed populations, and 4,396 females, accounting for 57.4% of all surveyed populations. The average age of the participants was 54.64 ± 13.26 years, and the average BMI was 25.36 ± 3.67. Most of the participants were of Han nationality, accounting for 98.7% in total. Most participants' education reached junior high school and high-school status, accounting for 55.8%. Most participants were married or living with partners, accounting for 93.0% of all surveyed participants. Between high intake and low intake groups, there were significant differences in gender, age, BMI, and education level (all *p* < 0.05). More details are available in Table 1.

**Table 1:** Characteristics of the participants (*n* = 7,665).

**Characteristics**	**All**	**High intake (>10.5 g/day)**	**Low intake (≤10.5 g/day)**	**t/χ^2^**	***P***
Gender, *n* (%)				124.98	<0.01
Male	3,269 (42.6)	2,537 (46.7)	732 (32.8)		
Female	4,396 (57.4)	2,896 (53.3)	1,500 (67.2)		
Age (years)	54.64 ± 13.26	55.55 ± 12.95	52.43 ± 13.74	9.16	<0.01
BMI (kg/m^2^)	25.36 ± 3.67	25.63 ± 3.69	24.70 ± 3.53	10.28	<0.01
Race				0.86	0.35
Han	7,566 (98.7)	5,367 (98.8)	2,199 (98.5)		
Other ethnicity	99 (1.3)	66 (1.2)	33 (1.5)		
Educational level				87.78	<0.01
Did not go to school	994 (13.0)	773 (14.2)	221 (9.9)		
Primary school	1,850 (24.1)	1,410 (26.0)	440 (19.7)		
Junior high school and high school	4,278 (55.8)	2,915 (53.7)	1,363 (61.1)		
Vocational college or undergraduate	553 (7.0)	327 (6.0)	206 (9.2)		
Postgraduate and above	10 (0.1)	8 (0.1)	2 (0.1)		
Marital status				4.10	0.25
Unmarried	177 (2.3)	116 (2.1)	61 (2.7)		
Getting married or cohabiting	7,127 (93.0)	5,056 (93.0)	2,071 (92.8)	
Divorce or separation	46 (0.6)	30 (0.6)	16 (0.7)	
Bereft of one's spouse	315 (4.1)	231 (4.3)	84 (3.8)	

### Salt-Reduction Knowledge, Behavior, and Salt Intake Status

Among the participants, only 1,481 (19.3%) had heard of the Dietary Guidelines for Chinese Residents. Their average salt intake was 11.89 ± 3.14 g/day, which was lower by 0.72 g/day than those who had not heard of the guidelines. Only 560 participants could correctly identify the recommended salt intake, accounting for 7.3% of all study populations. Their average salt intake was 11.76 ± 3.14 g/day, which was lower by 0.76 g/day than the participants who answered wrongly or who did not know what the recommended level was. The difference is statistically significant. Among the roughly 41% of participants who adopted different salt-reduction behaviors, the salt intake was 12.18 ± 3.17 g/day, while of the 59% of participants who adopted no salt-reduction behaviors, the salt intake was 12.67 ± 3.21 g/day. The salt intake of the participants who adopted any salt-reduction behavior was significantly lower than that of the participants who did not. More details are available in Table 2.

**Table 2:** Salt reduction knowledge, behavior, and salt intake status.

**Questions**	***n*** **(%)**	**Salt intake**	**t**	***P***
Have you heard of the dietary guidelines for Chinese residents?				
YES	1,481 (19.3)	11.89 ± 3.14	7.76	<0.01
NO	6,184 (80.7)	12.61 ± 3.20		
Did you know Chinese residents' dietary guidelines recommend daily salt intake for adults?				
Correct	560 (7.3)	11.76 ± 3.14	5.43	<0.01
Wrong or unknown	7,105 (92.7)	12.52 ± 3.20		
Whether salt reduction behaviors were taken				
YES	3,146 (41.0)	12.18 ± 3.17	6.45	<0.01
NO	4,519 (59.0)	12.67 ± 3.21		
Restrict consumption of processed foods				
YES	1,681 (21.9)	12.02 ± 3.17	−6.49	<0.01
NO	5,984 (78.1)	12.59 ± 3.20		
Note the salt on the food labels				
YES	919 (12.0)	11.95 ± 3.14	−5.25	<0.01
NO	6,746 (88.0)	12.54 ± 3.20		
Reduce eating out				
YES	2,010 (26.2)	12.03 ± 3.14	−7.11	<0.01
NO	5,655 (73.8)	12.62 ± 3.20		
Less salt when cooking				
YES	3,055 (39.86)	12.17 ± 3.17	−6.63	<0.01
NO	4,610 (60.14)	12.67 ± 3.21		
Eat less high-salt foods				
YES	2,321 (30.28)	12.11 ± 3.17	−6.52	<0.01
NO	5,344 (69.72)	12.63 ± 3.20		
No extra salt is added when eating at the table				
YES	2,081 (27.15)	12.03 ± 3.16	−7.41	<0.01
NO	5,584 (72.85)	12.63 ± 3.20		
Use salt-restriction spoon				
YES	1,039 (13.56)	12.10 ± 3.16	−4.02	<0.01
NO	6,626 (86.44)	12.53 ± 3.20		
Use low-sodium salt				
YES	795 (10.37)	11.93 ± 3.15	−5.03	<0.01
NO	6,870 (89.63)	12.53 ± 3.20		

### The Relationship Between Salt-Reduction Behaviors and Salt Intake

The study analyzed the association between salt-reduction behaviors and salt intake through logistic regression and MIV based on the BP neural network. In the logistic regression, it was only meaningful that “No extra salt was added at the table,” with the odds ratio (OR) being 0.78 (95% confidence interval [CI]: 0.64–0.95). Other variables were not included in the model. As is clear in **Table 4**, the result of MIV showed that the most important salt-reduction behavior was using “Less salt when cooking,” which was different from the logistic regression outcome. The second most important behavior was “No extra salt was added when eating,” while “Use salt-restriction spoon” and “Reduce eating out” were the least important. More details are available in Tables 3, 4.

**Table 3:** Logistic regression between salt reduction behaviors and salt intake.

**Variables**	**β**	**SE**	**Wald χ^2^**	***P***	**OR**	**95%CI**
Restrict consumption of processed foods	−0.03	0.09	0.07	0.79	0.96	(0.81, 1.17)
Note the salt of the food labels	−0.01	0.10	0.02	0.88	0.97	(0.82, 1.19)
Reduce eating out	−0.09	0.10	0.77	0.38	0.091	(0.75, 1.12)
Less salt when cooking food	−0.07	0.89	0.70	0.40	0.93	(0.78, 1.11)
Eat less high-salt foods	0.10	0.11	0.85	0.36	1.10	(0.89, 1.36)
No extra salt was added when eating at the table	−0.25	0.10	5.95	0.02	0.78	(0.64, 0.95)
Use salt-restriction spoon	−0.06	0.09	0.41	0.52	0.95	(0.80, 1.12)
Use low-sodium salt	−0.10	0.10	0.99	0.32	0.91	(0.75, 1.10)

**Table 4:** MIV between salt reduction behaviors and salt intake.

**Variables**	**Content**	**MIV**	**Rank number**
X1	Restrict consumption of processed foods	−0.0029	6
X2	Note the salt of the food labels	−0.0042	4
X3	Reduce eating out	−0.0024	7
X4	Less salt when cooking	−0.0236	1
X5	Eat less high-salt foods	0.0134	3
X6	No extra salt was added when eating at the table	−0.0227	2
X7	Use salt-restriction spoon	−0.0038	5
X8	Use low-sodium salt	0.0024	7

### BP Artificial Neural Networks Analysis

**Figure 1:**
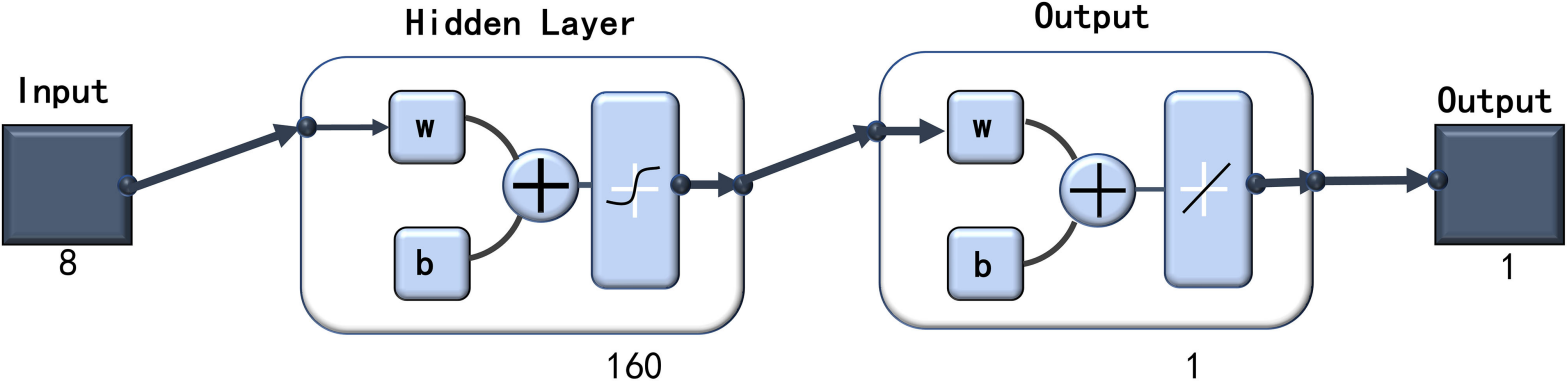
The configuration of the finel BPANN. The final establishment BPANN model consisted 1 input layer with 8 neurons (8 salt-reduction behaviors), 1 hidden layer with 160 nodes, and 1 output layer with 1 node (group of salt intake). BPANN indicates backpropagation artificial neural network.

Finally, the BP artificial neural network consisted of one input layer with 8 neurons, one hidden layer with 160 nodes, and one output layer with 1 neuron (Figure 1). The good fitness of the final network was apparent, indicated by the following three values: 0.2029 for mean squared error (MSE), 0.0022 for the magnitude of the gradient, and 0 for the number of validation checks. The network performance was considered to be a good pattern when the network contained eight risk factors (Table 4). According to the absolute value of MIV, the most important eight risk factors in rank order were as follows: less salt when cooking (MIV = −0.0236), no extra salt was added when eating at the table (MIV = −0.0227), eat less high salt foods (MIV = 0.0134), note the salt on the food labels (MIV = −0.0042), use salt-restriction spoon (MIV = −0.0038), restrict consumption of processed foods (MIV = −0.0029), reduce eating out (MIV = −0.0024), and use low-sodium salt (MIV = 0.0024) (Table 4).

## Discussion

This study showed the current status of salt-reduction awareness, salt-reduction behaviors, and salt intake of Chinese residents. It showed that only a small number of participants had salt intake-related knowledge or any awareness of salt-reduction behaviors. The salt intake of participants who adopted salt-reduction behaviors was significantly lower than that of participants who did not. In logistic regression and MIV, the effect of salt-reduction behaviors on salt intake was different. The study also showed that low-sodium salt and the salt-restriction spoon still needed better promotion. These results will inform the development of salt-reduction projects in China.

Only 560 participants, 7.3% of all survey populations, correctly identified the recommended salt intake level of the Chinese Dietary Guidelines. Similar to other studies, the vast majority of participants are not sure that they can recall or recognize the recommended salt intake level correctly (23–25). Their salt intake is significantly higher than that of participants who know the guidelines. This suggests that the promotion of nutrition and salt-related knowledge can help to reduce salt intake. Some studies believe that knowledge and behavior present a significant correlation (26); therefore, salt intake-related knowledge should be continuously promoted to raise the public's awareness of salt-reduction. Public awareness campaigns, which have always been one of the components of successful salt-reduction initiatives (27, 28), were among the several strategies recommended by the WHO for achieving population-based salt intake reduction. The awareness of the need for salt-reduction causes people to accept salt-reduction strategies to improve the health of the population and inclines them to adopt the corresponding salt reduction behaviors to actively reduce their own salt intake (29).

In the logistic regression results, only “No extra salt was added when eating at the table” had the effect of reducing salt intake. Unlike in Western countries, where much salt intake comes from processed foods (26, 30), the salt intake of Chinese residents mainly comes from the salt added to family cooking (31–33). The taste preference of Henan residents is for saltiness, although various salt-reduction campaigns have made Chinese residents aware of their source of salt and inclined them to reduce their family's salt addition during cooking (34). Unfortunately, for some people this means an unpalatable taste to their food, which means they will then add extra salt to their meal when eating. Only those who do not add extra salt can eventually reduce their salt intake. At the same time, 73.8% of participants chose to reduce their eating out, possibly due to the perception that the meals in restaurants are generally oily and salty, and that it is more difficult to control salt intake there compared with eating at home. Among people who adopted salt reduction behaviors, the percentage of participants who paid attention to food labels, used a salt-restriction spoon, or used low-sodium salt was <50%, respectively. This may be related to their inability to interpret the salt content information on food labels. Most food labels only indicate the sodium content, and most participants cannot accurately calculate the relationship between sodium and salt, and hence ignore food labels (35). Use of salt-restriction spoons and low-sodium salt are very effective ways to reduce salt intake in the population (33, 36). Low-sodium salt is a good substitute for salt, adds potassium, and reduces sodium intake. The small number of residents in Henan, China, who adopted these two behaviors indicates that more attention should be paid to promoting the use of salt-restriction spoons and low-sodium salt.

This study used logistic regression and the MIV based on the BP neural network to evaluate the association between salt-reduction behaviors and salt intake. In the logistic regression, “Only no extra salt was added when eating at the table” had the effect of reducing salt intake. It should be noted that the effect of this salt-reduction project is limited.This result may be related to the selected study population, or to dividing the groups by an intake of 10.5 g/day. MIV based on the BP neural network is a method that can be used to evaluate the importance of salt intake. With outcomes different from the logistic regression, the most important two factors were “Less salt when cooking food” and “No extra salt was added when eating at the table.” The results of these two variables were similar. The salt intake of Chinese residents mainly comes from cooking practices in the family, so the government and salt-reduction organizations should strengthen the salt intake propaganda related to family cooking. The last two factors in MIV were “Use low-sodium salt” and “Reduce eating out.” The result for “Use low-sodium salt” may be related to the small number of people adopting the behavior, such that its impact on overall salt intake is less than those behaviors widely adopted by the population. According to the results of other studies (36), however, the influence is obvious. Policymakers have already required salt-reduction project personnel to strengthen the promotion of salt-restriction tools and low-sodium salt (37), and to improve salt-reduction strategies in order to reach the salt-reduction goals of China and the WHO.

In this study, we used MIV to explore the importance of different salt-reduction behaviors and salt intake. The limitations of our study are mainly as follows. First, we used morning urine to estimate 24-hUNA instead of using the gold standard of multiple 24-hUNa to evaluate salt intake, which may attract some criticism. Second, our strategy of dividing the high-salt group and the low-salt group by 10.5 g/day instead of the 6 g/day recommended in China's dietary guidelines still needs validation.

## Conclusion

The popularization of salt-reduction knowledge and behaviors significantly reduces the salt intake of the population. In Henan, however, there is still considerable scope for the popularization of salt-reduction knowledge as there are still many people who have no awareness of the need for salt-reduction or of behaviors to achieve it.

## Data Availability Statement

The raw data supporting the conclusions of this article will be made available by the authors, without undue reservation.

## Ethics Statement

The studies involving human participants were reviewed and approved by Zhengzhou University Life Science Ethics Committee. The patients/participants provided their written informed consent to participate in this study.

## Author Contributions

BH: conceptualization and investigation. BH and CL: methodology and writing–original draft. YZ, MZ, CL, and TZ: data curation and formal analysis. YZ and DH: funding acquisition. LS, YZ, and DH: writing–review and editing. LS: supervision and project administration. All authors contributed to the article and approved the submitted version.

## Funding

This study was supported by the National Natural Science Foundation of China (grant no. 81973152).

## Conflict of Interest

The authors declare that the research was conducted in the absence of any commercial or financial relationships that could be construed as a potential conflict of interest.

## Publisher's Note

All claims expressed in this article are solely those of the authors and do not necessarily represent those of their affiliated organizations, or those of the publisher, the editors and the reviewers. Any product that may be evaluated in this article, or claim that may be made by its manufacturer, is not guaranteed or endorsed by the publisher.
